# Developing a Service Quality Index System for AI Health Care Chatbots: Mixed Methods Study

**DOI:** 10.2196/83051

**Published:** 2026-02-18

**Authors:** Yu Gu, Xinyi Wang

**Affiliations:** 1 School of Medical Technology Capital Medical University Beijing China

**Keywords:** artificial intelligence health care chatbot, artificial intelligence, AI, service quality, Delphi method, analytic hierarchy process, index system development, SERVQUAL

## Abstract

**Background:**

Artificial intelligence (AI) health care chatbots are gaining widespread adoption worldwide. It is imperative to understand the service quality of AI health care chatbots. However, there is limited guidance on how to comprehensively evaluate their service quality.

**Objective:**

This study aimed to develop an index system based on the SERVQUAL framework for evaluating the service quality of AI health care chatbots.

**Methods:**

An initial indicator pool was compiled through a comprehensive literature review and consultations with 4 experts. These indicators were mapped and categorized into 5 domains adapted from the SERVQUAL framework. The experts were recruited from hospital, university, and health commission settings by purposive sampling. The service quality index system was identified using a 2-round Delphi process, which included a virtual meeting between the 2 rounds. In the third round, indicator weights within each quality domain and subdomain were determined using the analytic hierarchy process.

**Results:**

There were 26 indicators identified in the literature, based on which the 2-round Delphi process was conducted. A total of 20 experts were invited. The response rates in both rounds of Delphi and the analytic hierarchy process were 100%, and the authoritative coefficients were both >0.7. The final service quality index system for AI health care chatbots comprises 5 primary indicators and 17 secondary indicators. There were 3 (18%) indicators on assurance, 4 (24%) on reliability, 3 (18%) on human-likeness, 4 (24%) on tangibility, and 3 (18%) on responsiveness. The primary indicators, ranked from highest to lowest weight, were assurance (0.239), reliability (0.237), human-likeness (0.187), tangibility (0.170), and responsiveness (0.167).

**Conclusions:**

This study pioneers the development of a service quality index system for AI health care chatbots adapted from the SERVQUAL framework. The results provide a validated tool for evaluating the performance of chatbots and offer valuable insights for health service managers and developers to enhance AI-driven medical consultation services.

## Introduction

Worldwide, artificial intelligence (AI) chatbots have been introduced into health care settings in recent years, where they are used by individuals as AI physicians for online medical consultations. A key innovation of AI health care chatbots lies in their ability to generate humanlike, natural language responses to diverse health-related queries anytime and anywhere, significantly improving access to medical guidance for broader populations [1]. Unlike earlier rule-based chatbots that relied on scripted replies, AI chatbots leverage advanced technologies, such as large language models (LLMs), to deliver personalized and context-aware interactions [2]. Moreover, the consultation service is often provided free of charge. AI health care chatbots show promise in delivering reliable medical advice without direct involvement from human physicians, offering a scalable solution to persistent challenges within the global health system, such as limited resources, uneven distribution, high costs, and growing demand [3]. Therefore, AI health care chatbots are playing an increasingly important role in modern health care systems [4].

AI health care chatbots represent not only a new type of service provider but also an innovative medical service model [5]. As an emerging field, chatbots have attracted growing attention from both practitioners and researchers. Despite its potential benefits, concerns remain regarding its service quality [6,7]. Efforts have been made to develop quality indicators for AI health care chatbots [8-16]. Some studies have evaluated response quality within specific disease contexts, such as labor epidurals, cardiovascular health, oncology, psoriasis, chronic hepatitis, and cancer [8-11]. Others have focused on assessing information quality [12,13] or have compared the performance of AI health care chatbots with that of human physicians [14-16]. However, existing studies primarily focus on narrow aspects of quality. Furthermore, the most commonly applied metrics—response accuracy, completeness, and consistency in closed-ended clinical questions—are predominantly defined from the health care providers’ perspective rather than that of users. Therefore, a comprehensive and user-centered index system for evaluating service quality of AI health care chatbots remains underdeveloped.

Among existing service quality frameworks, SERVQUAL, developed by Parasuraman et al [17], is one of the most widely recognized frameworks for evaluating medical service quality worldwide. This framework includes 5 dimensions—tangibility, reliability, responsiveness, assurance, and empathy—and is specifically designed to assess users’ expectations and perceptions of service quality [18]. Applying this classical framework enables a more comprehensive and theoretically grounded evaluation of service quality of AI health care chatbots, bridging classical service quality theory with emerging AI-driven health care contexts.

The aim of this study was to identify critical indicators that reflect the service quality of AI health care chatbots and to develop a scientifically feasible index system for its evaluation. The findings were expected to contribute to better identification of shortcomings, promote continuous quality improvement, enhance user experience, and offer new insights into the systemic evaluation of service quality of AI health care chatbots.

## Methods

### Study Design

**Figure 1:**
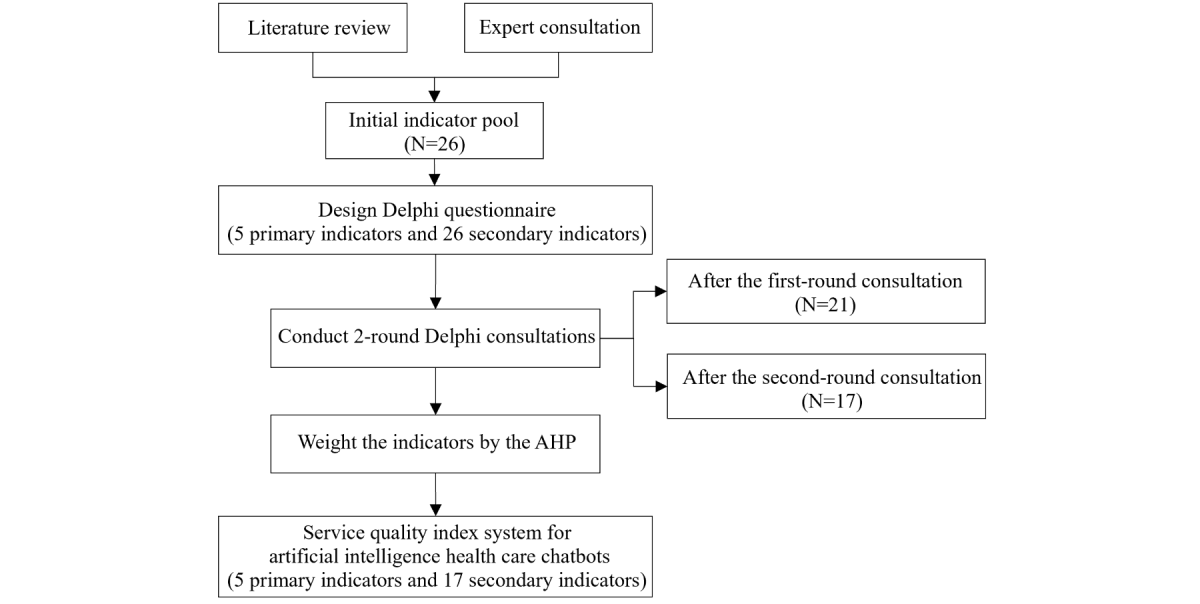
The research process. AHP: analytic hierarchy process.

This study used a mixed methods approach, combining qualitative insights from expert opinions with quantitative metrics to develop and quantify a service quality index system for AI health care chatbots. The literature review and expert consultation were applied to construct an initial indicator pool. The 2-round Delphi consultation was then conducted to refine and establish the final index system. Subsequently, the analytic hierarchy process (AHP) was applied to determine the weight of each indicator. The process of index system development and weight determination is shown in Figure 1.

### Initial Indicator Pool

The initial indicator pool was compiled based on existing literature and expert opinions. A comprehensive systematic literature search was conducted in 4 databases: PubMed, Web of Science, China National Knowledge Infrastructure, and Wanfang Data. The search strategy incorporated the following key terms: [“chatbot*” OR “chat-bot*” OR “conversational agent*” OR “conversational bot*” OR “conversational system*” OR “dialogue system*” OR ChatGPT] AND [“medic*” OR “health*” OR “disease*” OR “patient*”] AND [“quality indicator*” OR “quality evaluat*” OR “quality assess*” OR “quality measure*”]. Boolean operators (AND, OR, and NOT) were used to combine or refine search terms (Multimedia Appendix 1). The exclusion criteria were as follows: (1) studies not focused on the evaluation of AI health care chatbots; (2) commentaries, protocols, letters, editorials, and conference abstracts; and (3) studies not published in English or Chinese.

In the second phase, this study incorporated insights from 4 interdisciplinary experts specializing in intelligent health care and medical service management. These experts conducted in-depth discussions regarding the relevance, suitability, and validity of the preliminary indicators. None of them participated in the subsequent Delphi consultation rounds. Both prior literature and expert opinions emphasized that the SERVQUAL framework provides a user-centered foundation well suited for the evaluation of AI health care chatbots and highlighted anthropomorphism as a distinctive feature influencing perceived service quality in AI-driven interactions. Specifically, users desire AI health care chatbots to exhibit kindness through humanlike attributes, such as a name, image, and voice. Beyond a friendly appearance, users expect these systems to demonstrate social intelligence, including the ability to detect user emotions and respond with genuine concern, which goes beyond simple empathy. Furthermore, users expect personalized responses tailored to individual factors. Therefore, the *humanlike* dimension was introduced as an innovative replacement for the traditional “empathy” construct to better capture the emotional and interactive capabilities unique to AI health care chatbots. Accordingly, this study established 5 first-level indicators adapted from the SERVQUAL structure: tangibility, responsiveness, assurance, human-likeness, and reliability, and 26 second-level indicators were included in the initial pool.

### Expert Selection

The initial expert recruitment was conducted through recommendations from our collaborators in the field of intelligent health care. They were from renowned universities, tertiary hospitals, and provincial health sectors. To broaden the reach and ensure a diverse range of perspectives, the experts initially nominated by the researchers were then asked to suggest other qualified individuals who could contribute valuable insights to the study. The inclusion criteria for experts were as follows: (1) familiarity with research areas, such as intelligent health care, medical service management, health information management, and other related fields; (2) more than 5 years of professional experience in a relevant field; and (3) willingness to actively participate in the study and provide timely responses across multiple rounds of Delphi consultation. Finally, a total of 20 experts were recruited through purposive sampling.

### Delphi Process

The Delphi method is a structured communication technique designed to systematically collect expert opinions and achieve consensus [19]. It has been widely applied and validated as a robust research methodology in health care contexts [20]. This study conducted a 2-round Delphi consultation to screen, refine, and finalize the indicators.

The Delphi consultation questionnaire consists of 2 main sections: an informed consent form and the main survey. The informed consent form outlined the study’s background, objectives, methodology, privacy protection measures, and contact information. The main survey collected information from five areas: (1) experts’ basic information, including age, education, and years of work experience; (2) the core consultation content, in which experts scored the importance and feasibility of each indicator using a 10-point scale (1=lowest and 10=highest); (3) the familiarity scale, rated by the expert themselves using a 5-point Likert scale (1=very unfamiliar and 5=very familiar); (4) the basis of expert judgment, evaluating the impact of theoretical analysis, practical experience, literature knowledge, and instinct on scoring (rated as high, medium, or low); and (5) blank fields, allowing experts to propose additions, deletions, or modifications to the indicators. All experts completed the informed consent process, and strict confidentiality was maintained throughout the entire process.

The Delphi process was conducted between February 2025 and June 2025. In the first round, Delphi questionnaires in Microsoft Word format were distributed to 20 experts, with a 2-week response period. Experts were asked to rate both the primary and secondary indicators and to provide comments. On the basis of the results and comments from the first round, the questionnaire was revised and redistributed to the same 20 experts for the second round. The second round followed the same rating procedure as the first and achieved consensus among the experts.

### Indicator Selection

To screen the indicators, this study used 3 important statistics: the mean importance score, the full-mark rate (proportion of experts assigning the highest score), and the coefficient of variation. The inclusion criteria were as follows: (1) a mean of importance score ≥7.0, (2) a full-mark rate >20%, and (3) a coefficient of variation <0.25 [21-23]. Any indicator failing to meet all 3 criteria was subject to deletion or revision based on panel discussion and qualitative feedback.

### AHP Procedure

Following the 2-round Delphi consultation, the final set of indicators was confirmed. The same panel of experts was then invited to participate in a pairwise comparison process to determine indicator weights. For each pair of indicators within the same hierarchical level, judgment matrices were constructed using a 1 to 9 ordinal scale to assess their relative importance [24]. The weight of each indicator was subsequently calculated using the percentage weighting method based on the pairwise comparison matrices, with higher weight values indicating greater perceived importance.

### Data Analysis

Statistical analysis was conducted using SPSS software (version 25.0; IBM Corp). The authority coefficient (Cr) represents the authority level of experts. Cr was the arithmetic mean of the experts’ judgment coefficient (Ca) and the experts’ familiarity coefficient (Cs) [25]. A Cr value ≥0.7 was considered acceptable [26]. The Ca value was derived from experts’ self-assessment of their own judgment criteria, as detailed in Table 1. The Cs value ranges from 1.0 (very familiar) to 0.2 (unfamiliar). The coordination of expert opinions was tested using the Kendall coefficient of concordance (Kendall *W*), with a significance level of α=.05. YAAHP software (version 11.2; MetaDecision) was used to calculate the indicator weights and assess the consistency ratio. When the consistency ratio value was <0.10, it was considered acceptable, indicating sufficient consistency in expert judgments [27].

**Table 1:** The judgment basis and degree of influence.

Judgment basis	Degree of impact on experts’ judgment		
	High	Medium	Low
Theoretical analysis	0.3	0.2	0.1
Practical experience	0.5	0.4	0.3
Reference literature	0.1	0.1	0.1
Expert intuition	0.1	0.1	0.1

### Ethical Considerations

The study protocol was approved by the Ethics Committee of the Capital Medical University, Beijing, China (2025SY-071). Participants were informed of the study’s purpose and procedure. Online informed consent was obtained from each participant. All research data were stored on a password-encrypted computer, and only the researchers had access to the data. No compensation was provided to participants.

## Results

### Characteristics of Experts

A total of 20 experts completed the 2-round Delphi consultation and AHP evaluation. The panel consisted of 12 (60%) male and 8 (40%) female experts, ranging in age from 31 to 60 years. Among the experts, 17 (85%) held a master’s degree or higher. All experts possessed associate senior professional titles or higher. The panel included 10 (50%) experts from quality control departments of hospitals, 7 (35%) from universities, and 3 (15%) from national or regional health commissions. The detailed characteristics of these experts are summarized in Table 2.

**Table 2:** The characteristics of the Delphi consultation experts (N=20).

Characteristics		Experts, n (%)
**Sex**		
	Male	12 (60)
	Female	8 (40)
**Age (years)**		
	31-40	6 (30)
	41-50	11 (55)
	51-60	3 (15)
**Education**		
	Doctoral degree	10 (60)
	Master’s degree	7 (35)
	Bachelor’s degree	3 (15)
**Professional** **title**		
	Senior	11 (55)
	Associate senior	9 (45)
**Seniority (years)**		
	6-10	4 (20)
	11-20	9 (45)
	21-30	5 (25)
	>30	2 (10)
**Affiliation**		
	Hospitals	10 (50)
	Universities	7 (35)
	Health commission	3 (15)
**Field of expertise**		
	Intelligent health care	8 (40)
	Medical service management	7 (35)
	Health information management	5 (25)

### Authority Coefficient and Degree of Coordination

The Cr values for the first and second rounds of the Delphi consultation were 0.894 (Ca=0.931; Cs=0.847) and 0.919 (Ca=0.917; Cs=0.921), respectively (Table 3). Both values exceed the accepted threshold of 0.7, indicating a high level of expert credibility and reinforcing the reliability of the consultation results.

The Kendall *W* coefficients for the 2 consultation rounds are shown in Table 3. After the second round, the coordination coefficients of the indicator increased from 0.263 to 0.339, and all associated *P* values were <.001, indicating that the experts’ opinions converged and that the degree of consensus among experts was acceptable.

**Table 3:** Expert authority coefficients and the degree of coordination of expert opinions.

Round	Ca^a^	Cs^b^	Cr^c^	Kendall *W*	Chi-square (*df*)	*P* value
Round 1	0.931	0.847	0.894	0.263	71.1 (25)	<.001
Round 2	0.917	0.921	0.919	0.339	123.2 (20)	<.001

^a^Cs: familiarity coefficient.

^b^Ca: judgment coefficient.

^c^Cr: authority coefficient.

### Review for Initial Indicator Pool

**Figure 2:**
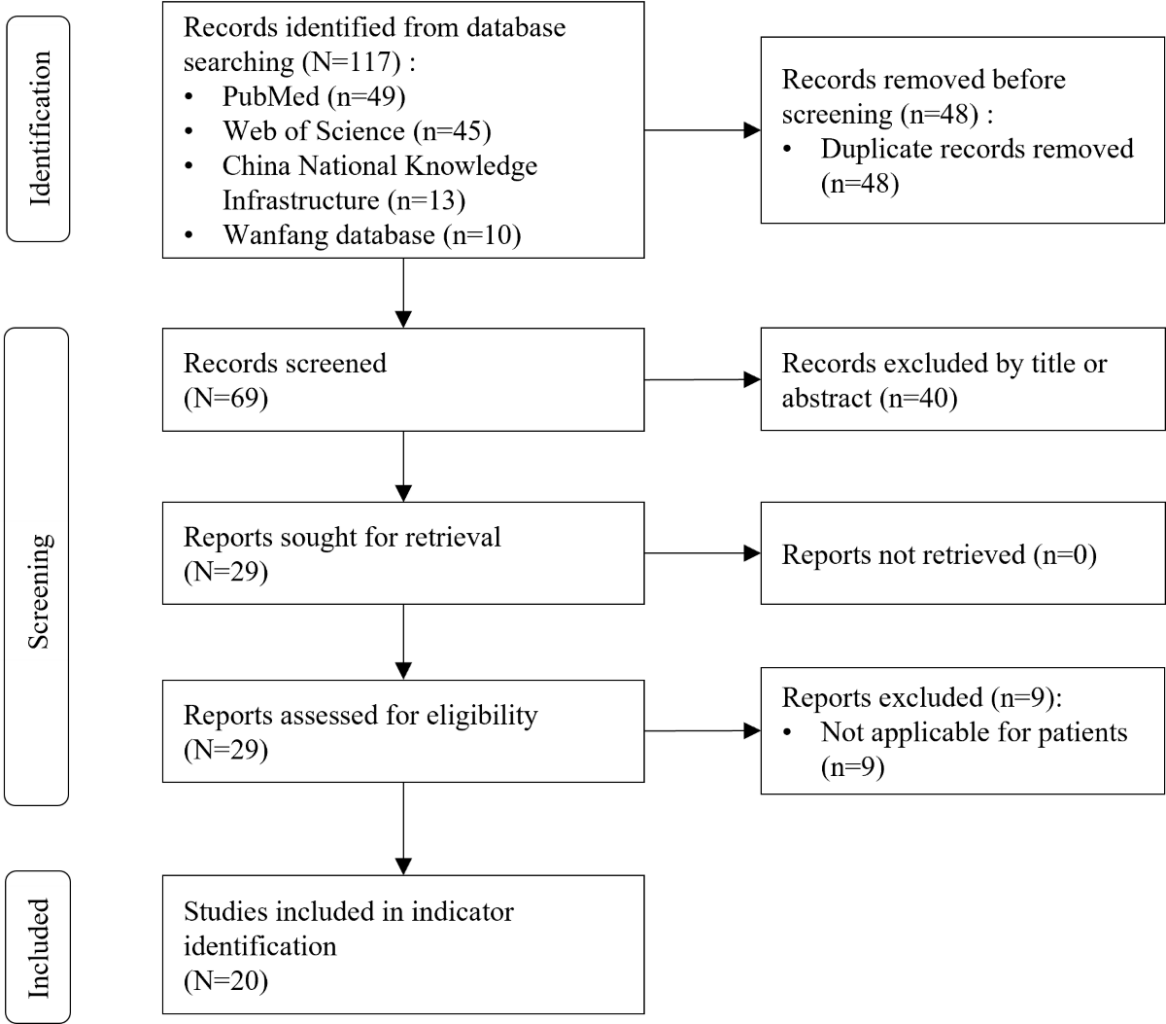
Flowchart of the included studies.

The database search and hand searches identified 117 articles, from which 48 (41.0%) duplicates were removed. After screening the titles and abstracts, 29 (24.8%) full-text records were reviewed, of which 20 (17.1%) were included in the review [28-47]. The study selection process is illustrated in Figure 2.

In the 20 eligible studies, 9 (45%) focused exclusively on AI chatbots designed specifically for health care service [28-36]. To develop a comprehensive initial indicator pool, we also included 11 additional studies concerning the quality of general AI chatbots capable of delivering health care–related consultations [37-47]. Of these, 6 (30%) studies aimed to develop instruments for measuring the overall service quality of AI chatbots [28,29,34,37,38,40], with 2 (10%) grounded in the SERVQUAL model [34,37]. Another 7 (35%) studies developed instruments targeting specific dimensions of quality [30-33,35,36,42]. Additionally, 7 (35%) studies treated quality as a key determinant of acceptance or satisfaction with AI chatbots and provided detailed measurement items for AI chatbot quality [39,41,43-47]. Following a systematic sorting process and discussions with 4 domain experts, we synthesized these findings into an initial pool of 26 second-level indicators for assessing the quality of AI health care chatbots, structured according to the 5 dimensions of the SERVQUAL framework.

### Indicator Selection

According to the indicator selection criteria and qualitative feedback from the experts, from a total of 26 indicators, 5 (19%) secondary indicators were removed and 21 (81%) were retained in the first round. Between round 1 and round 2, a virtual Delphi meeting was held to discuss indicators for which experts provided revision suggestions and to identify novel indicators based on perceived gaps in current indicators. Experts who had completed round 1 attended the meeting. During this panel meeting, 8 (31%) secondary indicators were merged into 4 (15%) indicators. Following the discussion, 20 experts rated the new indicators. Finally, the 2-round Delphi process reached a finalized evaluation framework comprising 5 (19%) primary indicators and 17 (65%) secondary indicators, each clearly defined in Table 4.

**Table 4:** The service quality index system for artificial intelligence (AI) health care chatbots.

Indicator		Definition	Weights
**Assurance**		The ability of AI health care chatbots to provide pertinent responses	0.239
	Understandable answer	The AI health care chatbots provide an answer with a logistical structure and the right amount of information to the user’s query	0.082
	Accurate understanding	The AI health care chatbots understand the exact meaning of the content sent by the user in text and voice	0.079
	Targeted question	The AI health care chatbots ask follow-up questions with context awareness based on the user’s query	0.078
**Reliability**		The ability of AI health care chatbots to inspire trust and confidence	0.237
	Trustworthy advice	The AI health care chatbots give medical advice, such as diagnosis, medication, and examination, and detailed explanations, which are consistent across multiple inquiries	0.071
	Useful service	The AI health care chatbots are useful in addressing users’ uncertainties about their health concerns	0.065
	Specific risk warning	The AI health care chatbots clearly indicate the limitations of its provided answers	0.050
	Protected privacy	The AI health care chatbots protect the user’s privacy	0.051
**Human-likeness**		The social cue, personality, and empathy of AI health care chatbots	0.187
	Personalized response	The AI health care chatbots tailor their answers according to the user’s age, sex, and medical history	0.064
	Emotional attention	The AI health care chatbots detect user emotion and makes the user feel concerned	0.063
	Kind characteristic	The AI health care chatbots have a kind name, image, and voice	0.060
**Tangibility**		The hardware or software manifestations of AI health care chatbots	0.170
	Accurate recognition	The AI health care chatbots recognize and transfer the speech of the users into accurate text	0.046
	Compatible operation	It is convenient for the user to obtain the service of AI health care chatbots on a mobile app, WeChat mini program, or website	0.043
	Friendly layout	The layout of AI health care chatbots is clear and easy to operate	0.041
	Stable service	The AI health care chatbots provide the same smooth service in any situation	0.040
**Responsiveness**		The response ability of AI health care chatbots	0.167
	Anytime response	The AI health care chatbots are available 24 hours a day for 365 days	0.043
	Prompt response	The AI health care chatbots always give timely feedback when it is needed	0.042
	Coherent response	The AI health care chatbots can communicate with the user seamlessly by maintaining records within the personal account	0.042

### Indicator Weights

On the basis of the AHP and percentage weighting method, the weights for all indicators were calculated (Table 4). The primary indicators, ranked from highest to lowest weight, were assurance (0.239), reliability (0.237), human-likeness (0.187), tangibility (0.170), and responsiveness (0.167). Assurance received the highest weight. For the secondary indicators, weights ranged from 0.040 to 0.082. “Understandable answer” had the highest secondary weight (0.082), followed by “Accurate understanding” (0.079), “Targeted question” (0.078), and “Trustworthy advice” (0.071).

## Discussion

### Principal Findings

The development of AI health care chatbots is on the rise, and their adoption is becoming increasingly vital in modern health care. Providing AI health care chatbots with high service quality is critical to facilitating their broader diffusion and addressing contemporary health care challenges. Although previous studies have attempted to evaluate the quality of AI chatbots in responding to queries related to specific diseases, a comprehensive and user-centered index system for evaluating the service quality of AI health care chatbots has remained lacking. To our knowledge, this is the first study to develop a comprehensive service quality index system for AI health care chatbots from a patient perspective, using SERVQUAL as the theoretical framework. Through a 2-round Delphi process, a finalized set of 5 primary indicators and 17 secondary indicators was derived, specifically designed to capture both the technical functionality and interactive experience unique to AI health care chatbots. Subsequently, the indicator weights were obtained using the AHP.

Among the 5 primary indicators, assurance was identified as the most important dimension, which refers to the ability of AI health care chatbots to provide pertinent responses. Unlike consultations with a clinician, AI health care chatbots lack the capacity to perform physical examinations to support diagnosis. Therefore, it is essential for AI health care chatbots to deliver goal-oriented and unambiguous conversations, accurately understand user queries, ask follow-up questions with contextual awareness, and provide understandable answers [39,48]. Previous studies [8-10] have taken understandability, often reflected through situation-appropriate response length and information quantity, as a sole metric to measure the quality of AI health care chatbots. Consistent with this emphasis, the secondary indicator “Understandable answer” received the highest weight among secondary indicators. Although aspects such as completeness and consistency have been associated with answer readability in previous studies [11,48], they were not included in this framework. This omission stems from their highly specialized and profession-centric evaluation criteria, which may not align with patient-centered usability expectations.

The reliability dimension ranked second, following assurance. Current AI technologies remain fallible and necessitate oversight by health professionals to ensure the applicability and safety advice of AI health care chatbots [7]. For users, it is critical that AI health care chatbots provide not only trustworthy medical advice regarding diagnosis, medication, and examinations but also clear explanations that enhance transparency and facilitate informed decision-making, thereby fostering trust and promoting sustained engagement [4,28]. Both users and clinicians have underscored the importance of clearly indicating the limitations of AI-generated medical advice [3-5]. In contrast to earlier studies [9,49], this study deliberately excluded diagnostic accuracy, a frequently used metric, from this index system, as patients generally lack the specialized medical knowledge required to evaluate this aspect.

The human-likeness dimension is considered a distinctive feature of AI health care chatbots [1]. Although it is not ranked highest among the primary indicators, its associated secondary indicators, “Personalized response” and “Emotional attention,” had prominent weight values. Users often perceive AI health care chatbots as an “AI doctor” and tend to evaluate it through direct comparison with human clinicians along these dimensions [3]. Enhancing humanlike attributes in interactions of AI health care chatbots remains a critical development objective. This entails increased efforts to enable AI health care chatbots to generate context-aware and individualized replies that adapt to both the conversational flow and user preferences [29].

The dimension of tangibility refers to hardware and software manifestations of AI health care chatbots. Among its secondary indicators, “Accurate recognition” was assigned the highest weight. As an information system designed to provide health guidance [50], the accuracy of recognizing and transferring user speech into precise text is the basis for correctly interpreting user queries and facilitating subsequent consultation processes [9]. The next important indicator, “Compatible operation,” reflects the accessibility of the service of AI health care chatbots across diverse digital environments. A previous study has emphasized that users valued the ability to access services of AI health care chatbots through various devices, such as smartphones, tablets, or computers, which supports broader and more equitable adoption [31].

Although responsiveness is positioned as the final dimension in the framework, it constitutes a fundamental component of the service quality of AI health care chatbots [5]. This dimension is characterized by the provision of active and uninterrupted guidance 24 hours a day, real-time responses without having to wait in line, immediate accessibility from any location without the need for travel, and seamless communication across different devices [21,28]. Given that AI health care chatbots are supported by LLMs, users perceive responsiveness as their inherent capability [1].

Overall, AI health care chatbots currently represent a viable alternative to human clinicians in initial user interactions [2]. However, its performance can vary significantly depending on the underlying LLMs, knowledge bases, and health data used [31]. While this study developed a service quality index system for AI health care chatbots based on the SERVQUAL framework, most secondary indicators in this study were newly developed to reflect the unique AI context. The proposed index system offers practical value for multiple stakeholders: it enables users to better understand and assess the strengths of AI health care chatbots; supports health service managers in systematically collecting feedback and monitoring performance; and guides developers in conducting feasibility analyses, optimizing design, and implementing postlaunch evaluation. Future research will involve applying this index system in field studies with users of AI health care chatbots to validate their utility and support its ongoing refinement.

### Strengths and Limitations

The strengths of this study are as follows. First, this study developed a comprehensive evaluation index system by adapting a scientific framework that incorporates both internationally validated evidence-based indicators and unique features of AI health care chatbots. Second, the mixed methods approach, combining literature review, expert consultations, a 2-round Delphi process, and AHP, ensured a rigorous and systematic development process. Third, this study provides new insights into the systemic evaluation of service quality of AI health care chatbots.

Several limitations should also be acknowledged. First, although the number of experts consulted met methodological requirements, it remained relatively limited. The panel may be subject to selection bias due to the experts’ familiarity with AI health care chatbots, which could influence the selection and weighting of indicators. Therefore, the scope of expert consultation needs to be further expanded to enhance the validity of the indicators. Second, all participating experts were based in China, which may limit the generalizability of the findings to other cultural or health system contexts. Future studies should validate the proposed evaluation index across a broader range of settings of AI health care chatbots. Third, this index system has not yet been operationalized and evaluated by the users of AI health care chatbots. Further empirical research is needed to demonstrate its practical relevance and utility and to consider incorporating patient experience into the assessment process. Furthermore, although Kendall *W* was statistically significant, its value reflects only a moderate level of consensus. This implies that the findings are robust but limited in microlevel ranking. In this study, experts from diverse professional backgrounds likely held different interpretations and assigned varying weights to the indicators, which may have led to evaluation discrepancies.

### Conclusions

This study developed a comprehensive, user-centered index system for evaluating the service quality of AI health care chatbots. Through the Delphi method and the AHP, a finalized framework consisting of 5 primary dimensions and 17 secondary indicators was established. These include 4 indicators for tangibility, 3 for responsiveness, 3 for assurance, 3 for human-likeness, and 4 for reliability. This index system prioritizes user needs and experiences and can practically quantify the service quality of AI health care chatbots. The proposed index system will provide valuable support for health policymakers, service managers, and developers by enabling benchmark comparisons, facilitating quality monitoring, and guiding continuous service enhancement.

## Data Availability

The datasets used or analyzed during this study are available from the corresponding author on reasonable request.

## Appendix

Multimedia Appendix 1 Search strategy.

## Abbreviations

AHP: analytic hierarchy process
AI: artificial intelligence
LLM: large language model
