# Supplementary material for: Developing a Service Quality Index System for AI Health Care Chatbots: Mixed Methods Study
Source: J Med Internet Res. 2026 Feb 18;28:e83051. doi: 10.2196/83051 (PMC12961388; doi:10.2196/83051)
Supplement: Multimedia Appendix 1 [file jmir_v28i1e83051_app1.docx]

**Search strategy**

PubMed:

(“chatbot*” [Title/Abstract] OR “chat-bot*” [Title/Abstract] OR “conversational agent*” [Title/Abstract] OR “conversational bot*” [Title/Abstract] OR “conversational” system* [Title/Abstract] OR “dialogue system *” [Title/Abstract] OR ChatGPT [Title/Abstract] ) AND ( “health*” [Title/Abstract] OR “medic*” [Title/Abstract] OR “disease*” [Title/Abstract] OR “patient*” [Title/Abstract]) AND (“quality indicator*” [Title/Abstract] OR “quality evaluat*” [Title/Abstract] OR “quality assess*” [Title/Abstract] OR “quality measure*” [Title/Abstract])

Web of science:

“chatbot*” OR “chat-bot*” OR “conversational agent*” OR “conversational bot*” OR “conversational” system*OR “dialogue system *” OR ChatGPT (Abstract) and “health*” OR “medic*” OR “disease*” OR “patient*” (Abstract) and “quality indicator*” OR “quality evaluat*” OR “quality assess*” OR “quality measure*” (Abstract)

China National Knowledge Infrastructure (CNKI):

TKA=(“医疗聊天机器人”+“医疗问答系统”+“ChatGPT”) AND TKA=(“服务质量”) AND TKA=(“评价”)

Wanfang database:

题名或关键词:(“医疗聊天机器人”+“医疗问答系统”+ “ChatGPT”) AND题名或关键词:(“服务质量”) AND题名或关键词:(“评价”)

Language limitations:

1.English

2.Chinese

Date limitations:

Published before January 2025
